# Supplementary material for: Finding cell-specific expression patterns in the early Ciona embryo with single-cell RNA-seq
Source: Sci Rep. 2020 Mar 18;10:4961. doi: 10.1038/s41598-020-61591-1 (PMC7080732; doi:10.1038/s41598-020-61591-1)
Supplement: Supplementary file 1 — Supplementary Figures. [file 41598_2020_61591_MOESM1_ESM.docx]

**Finding cell-specific expression patterns in the early Ciona embryo with single‑cell RNA-seq**

Garth R. Ilsley, Ritsuko Suyama, Takeshi Noda, Nori Satoh, Nicholas M. Luscombe

**Supplementary Figures and Tables**

Supplementary figures are in this file.

Supplementary tables can be found in the following files:

| **Supplementary Table 1** | List of known in situ patterns | Supplementary_Table_S1.xlsx |
| --- | --- | --- |
| **Supplementary Table 2** | Sampling, library preparation and sequencing  dates | Supplementary_Table_S2.xlsx |
| **Supplementary Table 3** | Sequencing statistics | Supplementary_Table_S3.xlsx |
| **Supplementary Table 4** | Primers used for qPCR | Supplementary_Table_S4.xlsx |
| **Supplementary Table 5** | IDs for the cDNA clones | Supplementary_Table_S5.xlsx |
| **Supplementary Table 6** | Single-cell qPCR measurements | Supplementary_Table_S6.xlsx |
| **Supplementary Table 7** | List of genes, their cluster patterns and TQR | Supplementary_Table_S7.xlsx |
